# Reversible aberrancies in gut microbiome of moderate and late preterm infants: results from a randomized, controlled trial

**DOI:** 10.1080/19490976.2023.2283913

**Published:** 2023-11-27

**Authors:** Raakel Luoto, Anna Pärtty, Josef K. Vogt, Samuli Rautava, Erika Isolauri

**Affiliations:** a Department of Pediatrics and Adolescent medicine, Turku University Hospital, Turku, Finland; b Institute of Clinical Medicine, University of Turku, Turku, Finland; c Clinical Microbiomics, Copenhagen, Denmark; d New Children’s Hospital, Helsinki University Hospital, Helsinki, Finland

**Keywords:** Bifidobacteria, gut microbiome, late preterm, moderate preterm, prebiotics, preterm, probiotics

## Abstract

The aim of this study was to obtain insight into the composition and function of the deviant gut microbiome throughout infancy in children born moderately and late preterm and their response to microbiome modulation. We characterized the longitudinal development of the gut microbiome from birth to the age of 12 months by metagenomic sequencing in 43 moderate and late preterm children participating in a randomized, controlled trial (ClinicalTrials.gov/no.NCT00167700) assessing the impact of a probiotic (*Lactobacillus rhamnosus* GG, ATCC 53,103, currently *Lacticaseibacillus rhamnosus GG*) and a prebiotic (galacto-oligosaccharide and polydextrose mixture, 1:1) intervention as compared to a placebo administered from 3 to 60 days of life. In addition, 9 full-term, vaginally delivered, breast-fed infants, who remained healthy long-term were included as references. Significant differences in taxonomy, but not in functional potential, were found when comparing the gut microbiome composition of preterm and full-term infants during the first month of life. However, the gut microbiome of preterm infants resembled that of full-term infants by 6 months age. Probiotic and prebiotic treatments were found to mitigate the shift in the microbiome of preterm infants by accelerating *Bifidobacteria*-dominated gut microbiome in beta diversity analysis. This study provides intriguing information regarding the establishment of the gut microbiome in children born moderately and late preterm, representing the majority of children born preterm. Specific pro- and prebiotics may reverse the proinflammatory gut microbiome composition during the vulnerable period, when the microbiome is low in resilience and susceptible to environmental exposure and simultaneously promotes immunological and metabolic maturation.

## Introduction

Preterm birth, defined as delivery prior to 37 weeks of gestation, affects approximately 10% of pregnancies worldwide and is the leading cause of neonatal morbidity and mortality.^1^ More than 80% of preterm children are born moderately (32 + 0–33 + 6) or late (34 + 0–36 + 6) preterm. The preterm gut microbiome profile, estimated soon after birth, differs from the full-term gut microbiome in terms of decreased diversity and a higher abundance of proinflammatory bacteria.^2–4^ Immediate perinatal exposures, including birth by cesarean section and perinatal and postnatal antibiotic treatment, as well as a lack of breast milk and parental skin-to-skin contact in the neonatal intensive care units (NICU), are factors that often cluster in preterm children, and may further disturb initial gut colonization.^5–9^

The compositional development of the gut microbiome of vaginally delivered full-term and breastfed children has been relatively well characterized while in the preterm counterpart, our understanding of the early stepwise microbiome establishment process remains incomplete.^10^ Specifically, the gap of knowledge involves the gut microbiome of moderate and late preterm children representing a majority of the infants born preterm.^5^ There is also a lack of follow-up data beyond the perinatal period throughout the stepwise compositional development of the gut microbiome. It is worth noting that there are current recommendations to modify the preterm gut microbiome using specific probiotics to fight acute health challenges. However, little is known about the impact of this on the microbiome *per se* both the short- and long-term.^11^

With these gaps in knowledge in mind, we prospectively characterized through metagenomic sequencing the compositional development of the gut microbiome of moderate and late preterm children during the first year of life and made comparisons with those of the full-term infants who remained healthy long-term, focusing on species composition and functional potential. We investigated whether the administration of specific probiotics or prebiotics might modulate the gut microbiome composition in moderate and late preterm infants and achieve colonization patterns resembling those in infants born full-term.

## Results

### Subject characteristics

**Figure 1.**
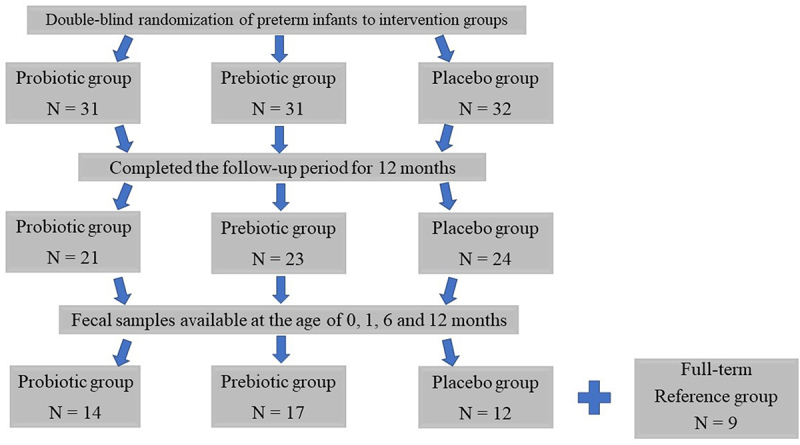
Trial flow of study infants.

The clinical characteristics of the preterm infants (*n* = 43) in the three intervention groups (Figure 1) and the full-term reference infants (*n* = 9) are presented in Table 1.

**Table 1.** Clinical characteristics of the full-term infants and the preterm infants in the three intervention groups.

Group	Full-term reference (*n* = 9)	Preterm Prebiotic (*n* = 17)	Preterm Probiotic (*n* = 14)	Preterm Placebo (*n* = 12)	*P* (a/b)
Gestational age (weeks)	40.1 (1.4)	34.5 (1.2)	35.2 (1.1)	34.7 (1.2)	NS/.000
Vaginal delivery (yes)	9 (100%)	11 (65%)	10 (71%)	7 (58%)	NS/NS
Antenatal corticosteroids (yes)	0 (0%)	12 (71%)	5 (36%)	5 (42%)	NS/.006
Intrapartum antibiotics (yes)	0 (0%)	4 (24%)	5 (36%)	5 (42%)	NS/NS
Sex (female)	6 (67%)	10 (59%)	4 (29%)	2 (17%)	.049/.038
Birth weight (g)	3483 (642)	2221 (392)	2600 (416)	2305 (455)	.046/.000
5-minute Apgar score	9 (1)	8 (3)	8 (2)	8 (1)	NS/NS
Need of NICU care	0 (0%)	13 (76%)	11 (79%)	9 (75%)	NS/.000
Number of days treated in NICU	-	13 (9)	8 (4)	12 (8)	NS/-
Postnatal antibiotic treatment	0 (0%)	9 (53%)	10 (71%)	8 (67%)	NS/.005
Length of postnatal antibiotic treatment (days)	0	1.8 (2.3)	2.3 (2.3)	1.8 (1.9)	NS/NS
Breastfed at the age of 1 mo (yes)	9 (100%)	17 (100%)	14 (100%)	11 (92 5)	NS/NS
Total length of breastfeeding (mo)	11.2 (5.9)	5.0 (2.4)	7.3 (4.7)	4.4 (4.7)	NS/.003
Antibiotic courses during the first 12 mo (yes)	0 (0%)	10 (59%)	13 (93%)	11 (92%)	.031/.000
Number of antibiotic courses during the first 12 mo (yes)*	0 (0%)	1.2 (1.5)	1.5 (1.0)	1.4 (1.1)	NS/.018

The data are presented as means (SD) for continuous variables and as numbers (percentage) for categorical variables.

The statistical analyses were made a) among the three preterm intervention groups and b) among the full-term and preterm study groups. The variables were analyzed using the χ2 test and Fisher´s exact test for dichotomous and analysis of variance (ANOVA) or Kruskal-Wallis test for continuous variables.

*Data informed by the parents.

NICU – neonatal intensive care unit.

### Comparison of the gut microbiome composition between preterm and full-term infants

We first characterized the compositional development of the gut microbiome throughout infancy in moderate and late preterm versus full-term infants by comparing the gut microbiome composition of preterm infants who had received a placebo (*n* = 4 at 0 months and *n* = 12 at 1, 6, and 12 months) to the gut microbiome composition of full-term, healthy, breastfed neonates (*n* = 9 at 0, 1, 6 and 12 months).

#### Alfa and beta diversity

**Figure 2.**
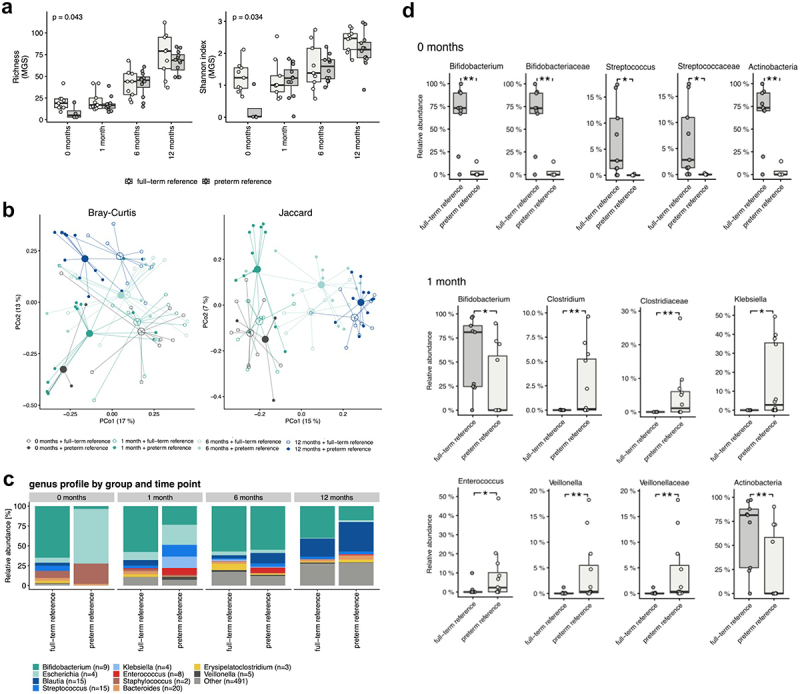
a) alpha diversity differences for preterm reference (placebo) and full-term reference groups at the age of 0, 1, 6, and 12 months represented as richness (MGS) and Shannon Index (MGS). P-values of the Mann-Whitney Y test are shown. Significant differences in alpha diversity were observed only at the age of 0 months (richness: *p* =.043, rank biserial correlation = −0.72; Shannon index: *p* =.034, rank biserial correlation = −0.78). b) beta diversity differences for the preterm reference (placebo) and full-term reference groups at the age of 0, 1, 6, and 12 months represented as a Bray–Curtis dissimilarity and a Jaccard Index calculation based on the metagenomic species (MGS) abundances. c) bar plot illustrating the taxonomic profiles aggregated at the genus levels and given separately for the preterm reference (placebo) and full-term reference groups at the age of 0, 1, 6, and 12 months. d) the significant taxa for preterm reference (placebo) and full-term reference groups at the age of 0 and 1 months. The results shown are for the Mann-Whitney U test FDR < 0.1 and the P-values of the Mann-Whitney U test (FDR <0.1).

Significantly lower alpha diversity was observed at the age of 0 months (i.e., sample taken within three days postpartum) among the preterm neonates who had received a placebo compared to the full-term neonates, as assessed by metagenomic species (MGS) richness (*p* =.043) and the Shannon index (*p* = .034) (Figure 2a). At the ages of 1, 6, and 12 months, no differences were detected in the alpha diversity between the two groups. The overall gut microbiome community structure of preterm infants who had received placebo and full-term infants followed a similar developmental trajectory (Figure 2b). However, the differences between these groups were found to be significant at the ages of 0, 1, and 12 months (*p* =.004, 0.003, and 0.040, respectively).

#### Taxonomy

In the first sample (0 months), the gut microbiome of preterm neonates was mainly composed of bacteria belonging to the phyla Firmicutes and Proteobacteria (Figure 2c). The two most abundant bacterial families in the initial preterm gut microbiome were *Enterobacteriaceae* and *Staphylococcaceae* (Figure 2c). In full-term neonates, at the phylum level, the gut microbiome in the first sample (0 months) was mainly composed of Actinobacteria, and the most abundant bacterial family was *Bifidobacteriaceae* (Figure 2c). At the age of 1 month, no significant changes compared to the initial phase were detected in the gut microbiome composition of preterm or full-term neonates. At the ages of 6 and 12 months, the gut microbiome composition of preterm infants resembled that of the full-term infants (Figure 2c).

#### Gut microbiota composition evolvement from birth to the age of 12 months

To determine the development of the gut microbiome composition throughout infancy, multiple hypothesis testing was used to compare the changes in gut microbiome composition between preterm infants who had received placebo and full-term infants. At the age of 0 months, the phylum Actinobacteria and its family *Bifidobacteriaceae* and its genus *Bifidobacterium* as well as the family *Streptococcaceae* and its genus *Streptococcus* were significantly lower in preterm infants than in full-term infants (false discovery rate, FDR < 0.1) (Figure 2d). At the age of 1 month, the relative abundance of the phylum Actinobacteria and the genus *Bifidobacterium* was significantly lower and that of the genera *Clostridium, Enterococcus, Klebsiella, Veillonella* was higher in preterm infants than in full-term infants (FDR <0.1) (Figure 2d). At the ages of 6 and 12 months, no significant taxonomic differences were found between preterm and full-term infants. No significant differences in microbiome gene-based functional potential were detected between the two groups at the ages of 0, 1, 6, and 12 months.

### The effect of probiotic and prebiotic intervention on the compositional development of preterm infant gut microbiome from birth throughout infancy

The second main focus of the present study was to assess the long-term impact of probiotic and prebiotic interventions on the compositional development of the gut microbiome in neonates born moderate and late preterm. The assessment was performed by comparing the gut microbiome of preterm infants receiving a probiotic, prebiotic, or placebo with each other and with full-term infants during the first 12 months of life.

#### Alfa and beta diversity

**Figure 3.**
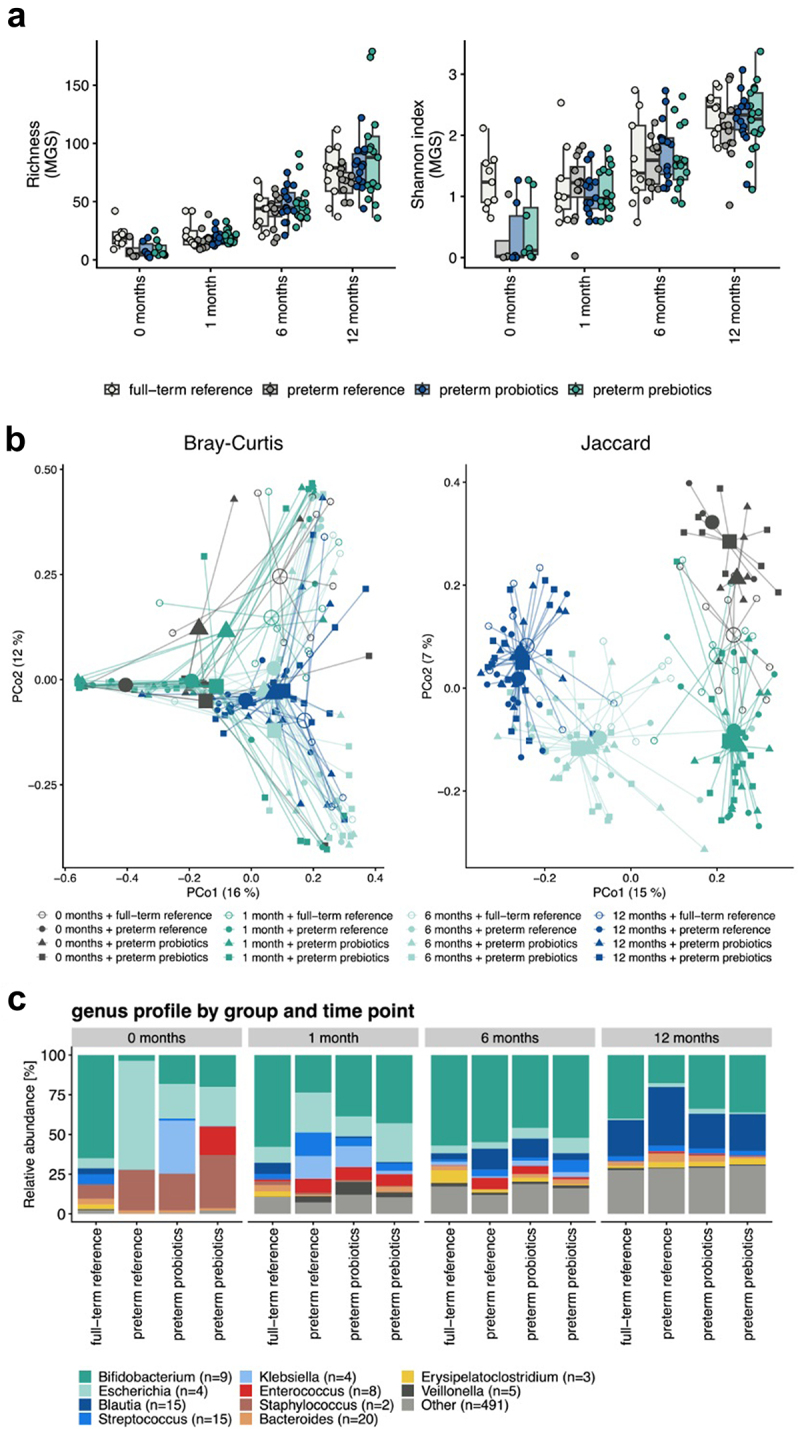
a) alpha diversity differences for the three preterm intervention groups (probiotics, prebiotics, and reference (placebo)) and full-term reference group at the age of 1, 6, and 12 months represented as richness (MGS) and Shannon Index (MGS). No significant differences were observed. b) beta diversity differences for the three preterm intervention groups (probiotics, prebiotics, and reference (placebo)) and full-term reference group at the age of 1, 6, and 12 months. The calculations are based on the MGS abundances and represented as Bray–Curtis dissimilarities and a Jaccard Index. c) bar plot illustrating the taxonomic profiles aggregated separately at the genus levels for the three preterm intervention groups (probiotics, prebiotics, and reference (placebo)) and full-term reference group at the age of 1, 6, and 12 months.

In this preterm study population, neither probiotic nor prebiotic intervention had a significant effect on gut microbiome alpha diversity at the ages of 1, 6, and 12 months (Figure 3a). However, beta diversity analysis indicated significant differences among the preterm study groups at 1 and 12 months of age (Figure 3b). When comparing the gut microbiome of the preterm infants in the three intervention groups to that of the full-term infants, significant differences in community structure were observed at the age of 1 month between the preterm reference (placebo) and preterm-prebiotic groups vs. the full-term reference group (*p* =.003 and 0.011, respectively), whereas the comparison between the preterm-probiotic and full-term reference groups was not statistically significant (*p* =.090). At the age of 6 months, no significant differences were detected among the study groups. At the age of 12 months, only the comparison between the preterm reference (placebo) and full-term reference groups was statistically significant (*p* =.040) (Figure 3b).

#### Taxonomy

In the first sample (0 months), the gut microbiome in all three preterm study groups was composed mainly of bacteria belonging to the phyla Firmicutes and Proteobacteria and the families *Enterobacteriaceae* and *Staphylococcaceae*. At the age of 1 month, in the gut microbiome of preterm infants receiving pro- and prebiotics, the most abundant phylum was Actinobacteria and family *Bifidobacteriaceae*, whereas in the gut microbiome of preterm infants receiving a placebo, Proteobacteria at the phylum level and *Enterobacteriaceae* at the family level dominated the gut microbiota. By the age of 6 months, the gut microbiome of infants in all three intervention groups resembled each other; in all groups, Actinobacteria was the dominant taxon at the phylum level and *Bifidobacteriaceae* at the family level. At the age of 12 months two different orders, *Bifidobacteriaceae* and *Clostridiales*, dominated the gut microbiome in all three intervention groups (Figure 3c).

#### Changes in microbiome composition

To assess whether gut microbiota modulation can revert the shift in the microbiome composition of preterm infants, the composition of MGSs and Gut Metabolic Modules (GMMs) of the placebo vs. probiotic and placebo vs. prebiotic groups were compared (data not shown). These analyses did not yield any significant hits with FDR < 0.1, except for MGS HG4C.2013: *Staphylococcus epidermidis* which was found to be significant for the interaction between treatment and time.

## Discussion

The results of the present study corroborate previous findings, according to which the early gut microbiome composition in children born preterm is distinct from that in children born full-term.^2,5,9,12–15^ Our results extend this finding to a longitudinal follow-up over the first year of life of moderate and late preterm infants, representing the majority of children born preterm. The follow-up period throughout infancy coincides with the age at which major changes occur in key regulatory processes of the body. We found that compared to healthy full-term infants, preterm infants´ gut colonization with *Bifidobacterium* was delayed, and as a substitute, their gut microbiome was enriched with *Escherichia* and *Streptococcus*. These changes were transient and amenable to microbiome modification by specific probiotics.

One in ten neonates are born prematurely, and approximately 85% of these are born moderately or late preterm, corresponding to approximately 12–13 million babies globally each year.^1^ Most research has been focused on extremely preterm infants since the frequency and severity of adverse outcomes are highest within this group. Moderate and late preterm infants exhibit markedly increased mortality and short- and long-term morbidity, including respiratory problems and asthma,^16–18^ neurodevelopmental and neurobehavioral impairment, and a need for special education.^19–23^ Many questions remain regarding the exact mechanisms underlying these risks, but interestingly, all these conditions have been linked to gut microbiome perturbations early in life.^8,24–26^

In general, the intestinal microbiome in preterm infants is characterized by delayed colonization, fewer bacterial species, less diversity, and an abundance of opportunistic and potentially pathogenic bacteria.^14,15,27,28^ When assessing the community structure in our study population, only in the first sample, taken within the first three days of life, were full-term infants detected as having a higher diversity than preterm infants. The difference was not detectable at later sampling points, indicating that independent of treatment, the shift in microbiome composition of preterm infants was, to some extent, already normalized at the age of one month. This finding is in line with that of a study by Jia and associates.^14^ However, differences in the taxonomic composition of the gut microbiome between full-term and preterm infants persisted longer. In full-term neonates, at the phylum level, the gut microbiome was mainly composed of the phylum *Actinobacteria*, the abundant bacterial family being *Bifidobacteriaceae*. In contrast, the gut microbiome of preterm infants randomized in the placebo group was found to be distinct from full-term infants; the unique features of their microbiome are the dominance of *Enterobacteriales* and *Staphylococcaceae* and the scarcity of *Bifidobacteriaceae*. The gut microbiome composition of full-term infants remains stable during the first six months of life. In contrast, the intestinal community structure of preterm infants converted to be more “term-like” during the first six months of life with the abundance of *Bifidobacteriaceae* increasing and that of *Enterobacteriales* decreasing. What is the clinical significance of this finding at individual level is naturally unclear. All these kinds of findings should be always interpreted with some caution, bearing in mind the limits of these reductionist approaches when considering the complex interactions between microbes, the host, and dietary and other environmental factors in the infant’s gastrointestinal tract.

The delayed colonization with *Bifidobacterium* and the abundance of potentially proinflammatory bacteria in the preterm gut microbiome during the first months of life in our study population was consistent with previously published studies.^5,13,29^ This finding is of considerable importance, as lower numbers of bifidobacteria in the infant intestine have previously been shown to be correlated with early life diseases such as necrotizing enterocolitis (NEC) and infantile colic, as well as later unfavorable sequelae such as atopic eczema, asthma, obesity, celiac disease, and other autoimmune diseases.^10,12,30–33^ It is well established that breastfeeding promotes a favorable gut microbiome structure and especially higher levels of *Bifidobacterium* species.^7,13,34^ In this study, all but one of the preterm infants were breastfed at the age of one month and the mean total duration of breastfeeding in these infants was 4.4 months. This might explain the observed progression of the preterm gut microbiome toward a *Bifidobacterium*-dominated composition occurring already at 6 months of age. Similarly, Korpela and associates provided evidence of an association between breastfeeding and “normal-like” microbiome development in a follow-up study with 45 very low birth weight (<1500 g) preterm infants.^13^

The differences in gut colonization patterns between full-term and preterm infants are often thought to be mediated by detrimental environmental exposures such as cesarean section delivery, shorter or no contact with the mother´s microbes at delivery, early antibiotic exposure, treatment in NICU environment, and longer hospitalization; with several of these factors often accumulating in preterm infants.^6–9^ Consistent with this, in our study population 35% of the preterm infants were born by cesarean section, 36% were exposed to intrapartum antibiotics, 75% were admitted to the NICU, 65% received antibiotics during the first days of life and 80% during their first year of life. Interestingly, we have previously shown that the differences in intestinal microbial colonization between full-term and late preterm infants can also be directly attributable to preterm birth *per se* and not only to detrimental environmental exposures.^5,35^ Although more mature than extremely preterm infants, infants born moderately and late preterm are also characterized by the immaturity of their immune regulation and gut barrier function, which may further selectively modulate microbial adhesion and colonization. In addition, the initial colonizing inoculum from the mother is dependent on the duration of pregnancy and may be further disturbed by either infection or preterm delivery *per se*.^36^ Nonetheless, the exact underlying mechanisms behind this deviant gut colonization remain elusive.

Alterations in the compositional development of the gut microbiome of newborns have been shown to predispose them to unfavorable conditions later in life. These are not only limited to short-term morbidities, including NEC and poor postnatal growth^3,8,29,37–39^ but may also manifest later in life as excessive weight gain and obesity, metabolic disease, allergies, and asthma.^39–42^ Impaired childhood growth is also known to be associated with poor neurodevelopmental outcomes^43,44^ and an increased risk for cardiometabolic diseases in later life.^45^

The undisputed impact of deviation in the early gut microbiome composition on later health calls for safe and effective modes of microbiome modulation.^10^ The results of this study demonstrate that this is achievable. In our study, the pro- and prebiotic treatment resulted in the mitigation of the early shift of the microbiome by accelerating *Bifidobacterium-*dominated gut microbiota composition during the first months of life. Notably, both pro- and prebiotic treatments modulated the preterm gut microbiome profile toward the “gold standard”, which is considered to be the microbiome of vaginally delivered, full-term, and breastfed children. This suggests that early probiotic and/or prebiotic intervention accelerated stepwise gut maturation processes in our preterm study population. Our previous studies demonstrated that early probiotic and prebiotic interventions might prevent eczema,^46^ infantile colic,^31^ and rhinovirus infections during the first year of life,^47^ highlighting the importance of early intervention. These observations are in line with programming theory, whereby early-life exposures, including microbial contact, may carry effects into later life, which is now being extended to microbial contact.^48,49^

The main strength of our study was the unique features of the full-term reference group with documented long-term health. The children in this reference group were born in the same hospital during the same period as the preterm population. The fact that 21 of the 206 fecal samples did not contain enough bacterial DNA and were excluded from further analysis may be considered a weakness of the study. It should be acknowledged that all the excluded samples were obtained from preterm subjects, which may reflect the delayed colonization of the gut microbiota in our preterm population compared to full-term infants.

Overall, the results of this study provide intriguing information about the establishment of the gut microbiome in moderately and late preterm infants throughout the first year of life and confirm the favorable effects of early probiotic and prebiotic interventions on gut microbiome maturation. In our study population, the compositional development of the gut microbiome and specifically colonization with *Bifidobacterium* in moderate and late preterm infants was delayed compared to that in full-term infants. The ground-breaking finding here is the transient nature of microbiome deviation in our moderate and late preterm infants. Indeed, much of the shift in microbiome composition had already spontaneously recovered during the first months of life, as assessed by alpha diversity. Consequently, deviation in the gut microbiome of these preterm infants could be characterized as a stability disturbance. According to current knowledge, deviant early microbial contact is not only involved in the initiation and perpetuation of aberrant immune activation and responsiveness^50,51^ but might underpin the central pathogenesis of a wide variety of conditions and diseases related to metabolic and immunological programming.^49,52,53^ Thus, modification of the initial gut microbiome could be a feasible tool to improve resilience against chronic diseases.

## Materials and methods

### Study population

The present study was based on a randomized, double-blind, placebo-controlled clinical trial (ClinicalTrials.gov/no.NCT00167700) described previously in greater detail.^31,47^ Briefly, 94 preterm neonates aged 1 to 3 days and treated at Turku University Hospital, Turku, Finland, between June 2008 and May 2012 were recruited. The inclusion criteria were as follows: gestational age between 32 + 0 and 36 + 6 weeks, birth weight greater than 1500 g, and absence of any congenital defects preventing enteral nutrition. During the first three days of life, the neonates were randomly assigned into one of the three study groups to receive the following: probiotic (*Lactobacillus rhamnosus* GG, ATCC 53,103; Mead Johnson & Co, Evansville, Ind., currently *Lacticaseibacillus rhamnosus GG*) at a dose of 1 × 10^9^ colony-forming units/day for intervention days 1 to 30 and at a dose of 2 × 10^9^ colony-forming units/day for intervention days 31 to 60, prebiotics (a mixture of polydextrose [Danisco Sweeteners, Surrey, United Kingdom] and galacto-oligosaccharides [Friesland Foods Domo, Zwolle, The Netherlands]) in a 1:1 ratio at a dose of 1 × 600 mg/day for intervention days 1 to 30 and at a dose of 2 × 600 mg/day for intervention days 31 to 60), or a placebo (microcrystalline cellulose and dextrose anhydrate [Chr. Hansen, Hørsholm, Denmark]).

Follow-up visits were scheduled at the ages of 1, 2, 4, 6, and 12 months by the same study nurse. During all study visits, the infants were clinically examined, and a detailed report was obtained from the parents of the infant’s behavioral patterns, to record any symptoms or signs of disease. The diagnostic protocols for respiratory tract infections and colic have been described in detail previously.^31,47^ From the original study population of 43 preterm infants (probiotic group, *n* = 14; prebiotic group, *n* = 17; placebo group, *n* = 12), fecal samples were obtained at the ages of 0, 1, 6, and 12 months, and these infants were included in the present analyses (Figure 1).

Healthy full-term reference subjects (*n* = 9) were selected from a randomized, double-blind, placebo-controlled clinical trial, which was conducted in the same hospital during the same period as the preterm trial.^54^ The control subjects fulfilled the following study criteria: randomization into the placebo group in the original trial, gestational age ≥ 37 + 0 weeks, vaginal delivery, breastfeeding, no intrapartum or postnatal antibiotic treatment or antibiotic courses during the first 12 months of age, and fecal sample availability at the age of 0, 1, 6, and 12 months.

This study complied with the Declaration of Helsinki, as revised in 2000. Written informed consent was obtained from the parents of the infants, and the study protocols were approved (11/2007 § 427) by the Ethics Committee of the Hospital District of South-West Finland.

### Sample collection

Fecal samples were collected from the diapers immediately after defecation. The first samples (meconium) were collected in the hospital by the study or NICU nurse within three days postpartum (i.e. 0 months sample). The timing of sample collection did not differ among the study groups. The later (1, 6 and 12 months) samples were collected by parents at home, immediately frozen to −20°C, and delivered to the study clinic within 24 hours. In the study clinic, the samples were frozen and stored at −80°C until analysis.

### DNA sequencing

Before sequencing, the quality of the DNA samples was evaluated using agarose gel electrophoresis and the quantity of the DNA was evaluated using Qubit 2.0 fluorometer quantitation. Genomic DNA was randomly sheared into fragments of approximately 350 base pairs. Fragmented DNA was used for library construction with the NEBNext Ultra Library Prep Kit for Illumina (New England Biolabs). The prepared DNA libraries were evaluated using Qubit 2.0, fluorometer quantitation, and Agilent 2100 Bioanalyzer for fragment size distribution. Quantitative real-time PCR (qPCR) was used to detect the final effective concentration with all samples with ≥ 2 nml library DNA were considered valid for library pooling. The library was sequenced using 2 × 150 bp paired-end sequencing on an Illumina platform.

### Gene catalog and definitions of the MGS

As a reference gene catalog, we used the Clinical Microbiomics Human Gut gene catalog (14,355,839 genes), which was created based on 12,170 nonpublic deep-sequenced human gut specimens (including 481 from infants), 9,428 publicly available metagenomes compiled from 43 countries^55^ and 3,567 publicly available genome assemblies from the isolated microbial strains. For taxonomic abundance profiling, a set of 2094 MGS was used, each represented by a set of genes with highly coherent abundance profiles and base compositions in the 12,170 metagenomes. The MGS concept was described by Nielsen et al. 2014.^56^

To taxonomically annotate an MGS, we blasted its genes against the NCBI RefSeq genome database (2020-01-27) and used the rank-specific annotation criteria. Specifically, we assigned a taxon to an MGS if at least M % of its genes were mapped to the taxon and no more than D % of its genes were mapped to a different taxon. We only considered blast hits with an alignment length of ≥ 100 bp, a query coverage ≥ 50%, and a % identity ≥ PID. Here we define: PID = (95, 95, 85, 75, 65, 55, 50, 45); *M* = (75, 75, 60, 50, 40, 30, 25, 20); and D = (10, 10, 10, 20, 20, 20, 20, 15) for subspecies, species, genus, family, order, class, phylum, and superkingdom, respectively. Finally, we processed each MGS with CheckM^57^ and updated our annotation with the CheckM result if this resulted in a lower taxonomic rank.

### Sequencing data preprocessing

Raw FASTQ files were filtered to remove host contamination by discarding read pairs in which either of the reads were mapped to the human reference genome GRCh38 with Bowtie2 (v. 2.3.4.1).^58^ Reads were trimmed to remove adapters and bases with a Phred score below 20 using an AdapterRemoval (v. 2.2.4).^59^ Read pairs in which both reads passed the filtering with a length of at least 100 bp were retained; these were classified as high-quality non-host (HQNH) reads. From the first samples, collected within three days postpartum (i.e. 0 months samples), 21 did not include bacterial DNA. These samples showed a high proportion of host DNA and a very low fraction of bacterial DNA. The latter can probably be interpreted as amplified noise. These samples were therefore excluded from further analysis. All excluded samples were from preterm infants.

### Mapping reads to the gene catalog

HQNH reads were mapped to the gene catalog using a BWA mem (v. 0.7.16a).^60^ An individual read was considered mapped to a gene if the mapping quality (MAPQ) was ≥ 20 and the read aligned with ≥ 95% identity over ≥ 100 bp. However, if > 10 bases of the read did not align with the gene or extend beyond the gene, the read was considered unmapped. Reads meeting the alignment length and identity criteria, but not the MAPQ threshold, were considered multi-mapped. Each read pair was counted as either 1) mapped to a specific gene, if one or both individual reads mapped to a gene; 2) multi-mapped, if neither read was mapped, and at least one was multi-mapped; or 3) unmapped, if neither individual read was mapped. If two reads mapped to a different gene, the gene mapped by read 1 was counted but not the gene mapped by read 2. A gene count table was created by using the number of mapped read pairs for each gene.

### MGS relative abundance calculation

For each MGS, a signature gene set was defined as 100 genes optimized for accurate abundance profiling. An MGS count table was created by counting the number of reads mapped to MGS signature genes per sample. An MGS was considered to be detected if reads from a sample mapped to at least three of its signature genes; measurements that did not satisfy this criterion were set to zero. Such a threshold resulted in 99.6% specificity. The MGS count table was normalized according to the effective gene length, and then normalized sample-wise to a sum of 100%, resulting in relative abundance estimates for each MGS.

Downsampled (rarefied) MGS abundance profiles were calculated by random sampling, without replacement, of a fixed number of signature gene counts per sample following the procedure described above. Samples that passed the initial QC and mapping were downsampled to 291,404 reads mapped to the signature genes.

### Diversity estimates

Alpha and beta diversity estimates were calculated from rarefied abundance matrices created by the random sampling of reads without replacement. Within each data type (e.g., gene, MGS), all samples were represented by the same number of informative sequencing reads: rarefaction of MGS abundance was performed by sampling only from reads mapped to the MGS signature genes, and rarefaction of Kyoto Encyclopedia of Genes and Genomes (KEGG) orthology (KO) abundance was performed by sampling only from reads mapped to a gene with an assigned KO. However, rarefaction of gene abundance was performed by sampling the reads mapped to the entire gene catalog. Alpha diversity was calculated as the number of entities detected (richness) or as the Shannon index based on a natural logarithm. Both measures were calculated from the downsized MGS abundance estimates. Beta diversity was calculated using Bray – Curtis dissimilarity and Jaccard index.

### Functional annotation and profiling

EggNOG-mapper (v. 2.0.1, Diamond mode)^61^ was used to map each gene in the gene catalog to the EggNOG (v. 5.0) orthologous groups database, resulting in EggNOG annotations for 79% of genes and KO database annotations for 46% of genes. Functional potential profiles based on KOs were calculated as the proportion of all mapped reads mapped to a given KO.

The GMMs are a set of 103 conserved metabolic pathways, each defined as a series of enzymatic steps represented by KO identifiers.^62^ We consider an MGS to contain a given module if the MGS included genes annotated to at least 2/3 of the KOs needed to complete the functionality of the module. If a module had alternative reaction paths, only one of them was required to be 2/3 complete. For modules with three or fewer steps, all steps must be included in the MGS.

### Statistical analysis

Statistical analyses for the baseline characteristics of the study population were performed using the χ2 test and Fisher´s exact test for dichotomous variables and an analysis of variance (ANOVA) or Kruskal-Wallis test for continuous variables, and data are presented as means (SD) for continuous variables and as numbers (percentage) for categorical variables.

To assess the changes in microbiome composition, the microbiome features (MGS, genus, family, phylum, and GMMs) of the full-term reference and preterm reference groups (preterm infants who had received placebo) were compared for each time point separately using the Mann-Whitney U test. To assess whether gut microbiome modulation can revert microbiome dysbiosis in preterm infants, a comparison was made between the composition of the MGSs and GMMs of the preterm reference vs. preterm prebiotic and the preterm reference vs. preterm probiotic.

When performing statistical testing on multiple hypotheses, the Benjamini – Hochberg (BH) method was used to control the FDR at a level of 10%, and a PERMANOVA was performed to test the community structure differences. This was done by using the adonis2 function from the vegan R package with 1000 permutations and by = “margin”, thus assessing the marginal effects of the terms (i.e. each marginal term was analyzed in a model with all the other variables).

## Data Availability

The datasets supporting the conclusions of this article are available from the European Nucleotide Archive (ENA) repository, Primary Accession: PRJEB62848 (https://www.ebi.ac.uk/).

## Abbreviations

FDR: False discovery rate
HQNH: high-quality non-host
GMM: Gut metabolic modules
MGS: Metagenomic species
NEC: Necrotizing enterocolitis
NICU: Neonatal intensive care unit

## Code availability

Source code for the randomization algorithms was from a previous study.^31,47^

## References

[1] Lawn JE, Ohuma EO, Bradley E, Idueta LS, Hazel E, Okwaraji YB, Erchick DJ, Yargawa J, Katz J, Lee AC, et al. Small babies, big risks: global estimates of prevalence and mortality for vulnerable newborns to accelerate change and improve counting. Lancet. 2023;401(10389):1707–14. doi:10.1016/S0140-6736(23)00522-6. 37167989

[2] Underwood MA, Sohn K. The microbiota of the extremely preterm infant. Clin Perinatol. 2017;44(2):407–427. doi:10.1016/j.clp.2017.01.005. 28477669 PMC6361543

[3] Hiltunen H, Hanani H, Luoto R, Turjeman S, Ziv O, Isolauri E, Salminen S, Koren O, Rautava S. Preterm infant meconium microbiota transplant induces growth failure, inflammatory activation, and metabolic disturbances in germ-free mice. Cell Rep Med. 2021;2(11):100447. doi:10.1016/j.xcrm.2021.100447. 34841294 PMC8606908

[4] Sim K, Powell E, Cornwell E, Kroll JS, Shaw AG. Development of the gut microbiota during early life in premature and term infants. Gut Pathog. 2023;15(1):3. doi:10.1186/s13099-022-00529-6. 36647112 PMC9841687

[5] Forsgren M, Isolauri E, Salminen S, Rautava S. Late preterm birth has direct and indirect effects on infant gut microbiota development during the first six months of life. Acta Paediatr. 2017;106(7):1103–1109. doi:10.1111/apa.13837. 28316118 PMC5763336

[6] Zou ZH, Liu D, Li HD, Zhu DP, He Y, Hou T, Yu JL. Prenatal and postnatal antibiotic exposure influences the gut microbiota of preterm infants in neonatal intensive care units. Ann Clin Microbiol Antimicrob. 2018;17(1):9. doi:10.1186/s12941-018-0264-y. 29554907 PMC5858143

[7] Stewart CJ, Ajami NJ, O´brien J, Hutchinson DS, Smith DP, Wong MC, Ross MC, Lloyd RE, Doddapaneni H, Metcalf GA, et al. Temporal development of the gut microbiome in early childhood from the TEDDY study. Nature. 2018;562(7728):583–588. doi:10.1038/s41586-018-0617-x. 30356187 PMC6415775

[8] Tirone C, Pezza A, Paladini A, Tana M, Aurilia C, Lio A, D’Ippolito S, Tersigni C, Posteraro B, Sanguinetti M, et al. Gut and lung microbiota in preterm infants: immunological modulation and implication in neonatal outcomes. Front Immunol. 2019;10:2910. doi:10.3389/fimmu.2019.02910. 31921169 PMC6920179

[9] Aguilar-Lopez M, Dinsmoor AM, Ho TTB, Donovan SM. A systematic review of the factors influencing microbial colonization of the preterm infant gut. Gut Microbes. 2021;13(1):1–33. doi:10.1080/19490976.2021.1884514. PMC8023245 33818293

[10] Milani C, Duranti S, Bottacini F, Casey E, Turroni F, Mahony J, Belzer C, Delgado Palacio S, Arboleya Montes S, Mancabelli L, et al. The first microbial colonizers of the human gut: composition, activities, and health implications of the infant gut microbiota. Microbiol Mol Biol Rev. 2017;81(4):e00036–17. doi:10.1128/MMBR.00036-17. PMC5706746 29118049

[11] Xiang Q, Yan X, Shi W, Li H, Zhou K. Early gut microbiota intervention in premature infants: application perspectives. J Adv Res. 2023;51:59–72. doi:10.1016/j.jare.2022.11.004. 36372205 PMC10491976

[12] Butel MJ, Suau A, Campeotto F, Magne F, Aires J, Ferraris L, Kalach N, Leroux B, Dupont C. Conditions of bifidobacterial colonization in preterm infants: a prospective analysis. J Pediatr Gastroenterol Nutr. 2007;44(5):577–582. doi:10.1097/MPG.0b013e3180406b20. 17460489

[13] Korpela K, Blakstad EW, Moltu SJ, Strømmen K, Nakstad B, Rønnestad AE, Brække K, Iversen PO, Drevon CA, de Vos W. Intestinal microbiota development and gestational age in preterm neonates. Sci Rep. 2018;8(1):2453. doi:10.1038/s41598-018-20827-x. 29410448 PMC5802739

[14] Jia J, Xun P, Wang X, He K, Tang Q, Zhang T, Wang Y, Tang W, Lu L, Yan W, et al. Impact of postnatal antibiotics and parenteral nutrition on the gut microbiota in preterm infants during early life. J Parenter Enter Nutr. 2020;44(4):639–654. doi:10.1002/jpen.1695. 31452218

[15] Chi C, Fan Y, Li C, Li Y, Guo S, Li T, Buys N, Clifton VL, Colditz PB, Yin C, et al. Early gut microbiota colonisation of premature infants fed with breastmilk or formula with or without probiotics: a cohort study. Nutrients. 2021;13(11):4068. doi:10.3390/nu13114068. 34836323 PMC8624512

[16] Drysdale SB, Lo J, Prendergast M, Alcazar M, Wilson T, Zuckerman M, Smith M, Broughton S, Rafferty GF, Peacock JL, et al. Lung function of preterm infants before and after viral infections. Eur J Pediatr. 2014;173(11):1497–1504. doi:10.1007/s00431-014-2343-1. 24898777

[17] Haataja P, Korhonen P, Ojala R, Hirvonen M, Paassilta M, Gissler M, Luukkaala T, Tammela O. Asthma and atopic dermatitis in children born moderately and late preterm. Eur J Pediatr. 2016;175(6):799–808. doi:10.1007/s00431-016-2708-8. 26898703

[18] Crump C, Sundquist J, Sundquist K. Preterm or early term birth and long-term risk of asthma into midadulthood: a national cohort and cosibling study. Thorax. 2023;78(7):653–660. doi:10.1136/thorax-2022-218931. 35907641 PMC9884998

[19] Chawanpaiboon S, Vogel JP, Moller AB, Lumbiganon P, Petzold M, Hogan D, Landoulsi S, Jampathong N, Kongwattanakul K, Laopaiboon M, et al. Global, regional, and national estimates of levels of preterm birth in 2014: a systematic review and modelling analysis. Lancet Glob Health. 2019;7(1):e37–e46. doi:10.1016/S2214-109X(18)30451-0. 30389451 PMC6293055

[20] Jamaluddine Z, Sharara E, Helou V, El Rashidi N, Safadi G, El-Helou N, Ghattas H, Sato M, Blencowe H, Campbell OMR. Effects of size at birth on health, growth and developmental outcomes in children up to age 18: an umbrella review. Arch Dis Child. 2023 Jun 20. archdischild-2022–324884. Online ahead of print. doi:10.1136/archdischild-2022-324884. PMC11474254 37339859

[21] Harijan P, Boyle EM. Health outcomes in infancy and childhood of moderate and late preterm infants. Semin Fetal Neonatal Med. 2012;17(3):159–162. doi:10.1016/j.siny.2012.02.002. 22417643

[22] Boyle JD, Boyle EM. Born just a few weeks early: does it matter? Arch Dis Child Fetal Neonatal Ed. 2013;98(1):F85–88. doi:10.1136/archdischild-2011-300535. 21865487

[23] Natarajan G, Shankaran S. Short- and long-term outcomes of moderate and late preterm infants. Am J Perinatol. 2016;33(3):305–317. doi:10.1055/s-0035-1571150. 26788789

[24] Sbihi H, Boutin RCT, Cutler C, Suen M, Finlay BB, Turvey SE. Thinking bigger: how early-life environmental exposures shape the gut microbiome and influence the development of asthma and allergic disease. Allergy. 2019;74(11):2103–2115. doi:10.1111/all.13812. 30964945

[25] Heiss CN, Olofsson LE. The role of the gut microbiota in development, function and disorders of the central nervous system and the enteric nervous system. J Neuroendocrinol. 2019;31(5):12684. doi:10.1111/jne.12684. 30614568

[26] Cryan JF, O´riordan OJ, Cowan CSM, Sandhu KV, Bastiaanssen TFS, Boehme M, Codagnone MG, Cussotto S, Fulling C, Golubeva AV, et al. The microbiota-gut-brain axis. Physiol Rev. 2019;99(4):1877–2013. doi:10.1152/physrev.00018.2018. 31460832

[27] Henderickx JGE, Zwittink RD, van Lingen RA, Knol J, Belzer C. The preterm gut microbiota: an inconspicuous challenge in nutritional neonatal care. Front Cell Infect Microbiol. 2019;9:85. doi:10.3389/fcimb.2019.00085. 31001489 PMC6454191

[28] Cuna A, Morowitz MJ, Ahmed I, Umar S, Sampath V. Dynamics of the preterm gut microbiome in health and disease. Am J Physiol Gastrointest Liver Physiol. 2020;320(4):G411–G419. doi:10.1152/ajpgi.00399.2020. PMC8238167 33439103

[29] Arboleya S, Martinez-Camblor P, Solís G, Suárez M, Fernández N, de Los Reyes-Gavilán CG, Gueimonde M. Intestinal microbiota and weight-gain in preterm neonates. Front Microbiol. 2017;8:183. doi:10.3389/fmicb.2017.00183. 28228752 PMC5296308

[30] Kalliomäki M, Collado MC, Salminen S, Isolauri E. Early differences in fecal microbiota composition in children may predict overweight. Am J Clin Nutr. 2008;87(3):534–538. doi:10.1093/ajcn/87.3.534. 18326589

[31] Pärtty A, Luoto R, Kalliomäki M, Salminen S, Isolauri E. Effects of early prebiotic and probiotic supplementation on development of gut microbiota and fussing and crying in preterm infants: a randomized, double-blind, placebo-controlled trial. J Pediatr. 2013;163(5):1272–1277.e1–2. doi:10.1016/j.jpeds.2013.05.035. 23915796

[32] Tojo R, Suarez A, Clemente MG, de Los Reyes-Gavilán CG, Margolles A, Gueimonde M, Ruas-Madiedo P. Intestinal microbiota in health and disease: role of bifidobacteria in gut homeostasis. World J Gastroenterol. 2014;20(14):15163–15176. doi:10.3748/wjg.v20.i41.15163. 25386066 PMC4223251

[33] Stewart CJ, Nelson A, Treumann A, Skeath T, Cummings SP, Embleton ND, Berrington JE. Metabolomic and proteomic analysis of serum from preterm infants with necrotising entercolitis and late-onset sepsis. Pediatr Res. 2016;79(3):425–431. doi:10.1038/pr.2015.235. 26571220 PMC4823643

[34] Ames SR, Lotoski LC, Azad MB. Comparing early life nutritional sources and human milk feeding practices: personalized and dynamic nutrition supports infant gut microbiome development and immune system maturation. Gut Microbes. 2023;15(1):2190305. doi:10.1080/19490976.2023.2190305. 37055920 PMC10114993

[35] Hiltunen H, Collado MC, Ollila H, Kolari T, Tölkkö S, Isolauri E, Salminen S, Rautava S. Spontaneous preterm delivery is reflected in both early neonatal and maternal gut microbiota. Pediatr Res. 2022;91(7):1804–1811. doi:10.1038/s41390-021-01663-8. 34349229 PMC9270225

[36] Koren O, Goodrich JK, Cullender TC, Spor A, Laitinen K, Bäckhed HK, Gonzalez A, Werner JJ, Angenent LT, Knight R, et al. Host remodeling of the gut microbiome and metabolic changes during pregnancy. Cell. 2012;150(3):470–480. doi:10.1016/j.cell.2012.07.008. 22863002 PMC3505857

[37] Neu J, Walker WA. Necrotizing enterocolitis. N Engl J Med. 2011;364(3):255–264. doi:10.1056/NEJMra1005408. 21247316 PMC3628622

[38] Ruiz L, Moles L, Gueimonde M, Rodriguez JM. Perinatal microbiomes’ influence on preterm birth and preterms’ health: influencing factors and modulation strategies. J Pediatr Gastroenterol Nutr. 2016;63(6):e193–e203. doi:10.1097/MPG.0000000000001196. 27019409

[39] Uzan-Yulzari A, Turta O, Belogolovski A, Ziv O, Kunz C, Perschbacher S, Neuman H, Pasolli E, Oz A, Ben-Amram H, et al. Neonatal antibiotic exposure impairs child growth during the first six years of life by perturbing intestinal microbial colonization. Nat Commun. 2021;12(1):443. doi:10.1038/s41467-020-20495-4. 33500411 PMC7838415

[40] Cox LM, Yamanishi S, Sohn J, Alekseyenko AV, Leung JM, Cho I, Kim SG, Li H, Gao Z, Mahana D, et al. Altering the intestinal microbiota during a critical developmental window has lasting metabolic consequences. Cell. 2014;158(4):705–721. doi:10.1016/j.cell.2014.05.052. 25126780 PMC4134513

[41] Hu T, Dong Y, Yang C, Zhao M, He Q. Pathogenesis of children´s allergic diseases: refocusing the role of the gut microbiota. Front Physiol. 2021;12:749544. doi:10.3389/fphys.2021.749544. 34721073 PMC8551706

[42] Yao Y, Cai X, Ye Y, Wang F, Chen F, Zheng C. The role or microbiota in infant health: from early life to adulthood. Front Immunol. 2021;12:708472. doi:10.3389/fimmu.2021.708472. 34691021 PMC8529064

[43] Dotinga BM, Eshuis MS, Bocca-Tjeertes IF, Kerstjens JM, Van Braeckel KN, Reijneveld SA, Bos AF. Longitudinal growth and neuropsychological functioning at age 7 in moderate and late preterms. Pediatrics. 2016;138(4):e20153638. doi:10.1542/peds.2015-3638. 27940890

[44] De Nardo MC, Mario CD, Laccetta G, Boscarino G, Terrin G. Enteral and parenteral energy intake and neurodevelopment in preterm infants: a systematic review. Nutrition. 2021;97:111572. doi:10.1016/j.nut.2021.111572. 35306422

[45] De Lucia Rolfe E, de Franga GVA, Vianna CA, Gigante DP, Miranda JJ, Yudkin JS, Horta BL, Ong KK. Associations of stunting in early childhood with cardiometabolic risk factors in adulthood. PLoS ONE. 2018;13(4):e0192196. doi:10.1371/journal.pone.0192196. 29641597 PMC5894958

[46] Kalliomäki M, Salminen S, Arvilommi H, Kero P, Koskinen P, Isolauri E. Probiotics in primary prevention of atopic disease: a randomised placebo-controlled trial. Lancet. 2001;357(9262):1076–1079. doi:10.1016/S0140-6736(00)04259-8. 11297958

[47] Luoto R, Ruuskanen O, Waris M, Kalliomäki M, Salminen S, Isolauri E. Prebiotic and probiotic supplementation prevents rhinovirus infections in preterm infants: a randomized, placebo-controlled trial. J Allergy Clin Immunol. 2014;133(2):405–413. doi:10.1016/j.jaci.2013.08.020. 24131826 PMC7112326

[48] Faith JJ, Guruge JL, Charbonneau M, Subramanian S, Seedorf H, Goodman AL, Clemente JC, Knight R, Heath AC, Leibel RL, et al. The long-term stability of the human gut microbiota. Sci. 2013;341(6141):1237439. doi:10.1126/science.1237439. PMC3791589 23828941

[49] Gensollen T, Iyer SS, Kasper DL, Blumberg RS. How colonization by microbiota in early life shapes the immune system. Sci. 2016;352(6285):539–544. doi:10.1126/science.aad9378. PMC5050524 27126036

[50] Hooper LV, Littman DR, Macpherson AJ. Interaction between the microbiota and immune system. Sci. 2012;336(6086):1268–1273. doi:10.1126/science.1223490. PMC4420145 22674334

[51] Olszak T, An D, Zeissig S, Vera MP, Richter J, Franke A, Glickman JN, Siebert R, Baron RM, Kasper DL, et al. Microbial exposure during early life has persistent effects on natural killer T cell function. Sci. 2012;336(6080):489–493. doi:10.1126/science.1219328. PMC3437652 22442383

[52] Gluckman PD, Hanson MA, Cooper C, Thornburg KL. Effect of in utero and early-life conditions on adult health and disease. N Engl J Med. 2008;359(1):61–73. doi:10.1056/NEJMra0708473. 18596274 PMC3923653

[53] Rautava S, Luoto R, Salminen S, Isolauri E. Microbial contact during pregnancy, initial colonization and human disease. Nat Rev Gastroenterol Hepat. 2012;9(10):565–576. doi:10.1038/nrgastro.2012.144. 22890113

[54] Rautava S, Kainonen E, Salminen S, Isolauri E. Maternal probiotic supplementation during pregnancy and breast-feeding reduces the risk of eczema in the infant. J Allergy Clin Immunol. 2012;130(6):1355–1360. doi:10.1016/j.jaci.2012.09.003. 23083673

[55] Pasolli E, Asnicar F, Manara S, Zolfo M, Karcher N, Armanini F, Beghini F, Manghi P, Tett A, Ghensi P, et al. Extensive unexplored human microbiome diversity revealed by over 150,000 genomes from metagenomes spanning age, geography, and lifestyle. Cell. 2019;176(3):649–662.e20. doi:10.1016/j.cell.2019.01.001. 30661755 PMC6349461

[56] Nielsen HB, Almeida M, Juncker AS, Rasmussen S, Li J, Sunagawa S, Plichta DR, Gautier L, Pedersen AG, Le Chatelier E, et al. Identification and assembly of genomes and genetic elements in complex metagenomic samples without using reference genomes. Nat Biotechnol. 2014;32(8):822–828. doi:10.1038/nbt.2939. 24997787

[57] Parks DH, Imelfort M, Skennerton CT, Hugenholtz P, Tyson GW. CheckM: assessing the quality of microbial genomes recovered from isolates, single cells, and metagenomes. Genome Res. 2015;25(7):1043–1055. doi:10.1101/gr.186072.114. 25977477 PMC4484387

[58] Langmead B, Salzberg SL. Fast gapped-read alignment with bowtie 2. Nat Methods. 2012;9(4):357–359. doi:10.1038/nmeth.1923. 22388286 PMC3322381

[59] Schubert M, Lindgreen S, Orlando L. AdapterRemoval v2: rapid adapter trimming, identification, and read merging. BMC Res Notes. 2016;9(1):88. doi:10.1186/s13104-016-1900-2. 26868221 PMC4751634

[60] Li H, Durbin R. Fast and accurate short alignment with Burrows-Wheeler transform. Bioinformatics. 2009;25(14):1754–1760. doi:10.1093/bioinformatics/btp324. 19451168 PMC2705234

[61] Huerta-Cepas J, Forslund K, Coelho LP, Szklarczyk D, Jensen LJ, von Mering C, Bork P. Fast genome-wide functional annotation through orthology assignment by eggNOG-Mapper. Mol Biol Evol. 2017;34(8):2115–2122. doi:10.1093/molbev/msx148. 28460117 PMC5850834

[62] Vieira-Silva S, Falony G, Darzi Y, Lima-Mendez G, Garcia Yunta R, Okuda S, Vandeputte D, Valles-Colomer M, Hildebrand F, Chaffron S, et al. Species-function relationships shape ecological properties of the human gut microbiome. Nature Microbiology. 2016;1(8):16088. doi:10.1038/nmicrobiol.2016.88. 27573110

